# Family Planning Beliefs and Their Association with Contraceptive Use Dynamics: Results from a Longitudinal Study in Uganda

**DOI:** 10.1111/sifp.12153

**Published:** 2021-05-20

**Authors:** Linnea A. Zimmerman, Dana O. Sarnak, Celia Karp, Shannon N. Wood, Caroline Moreau, Simon P. S Kibira, Fredrick Makumbi

**Affiliations:** ^1^ Department of Population, Family and Reproductive Health Johns Hopkins Bloomberg School of Public Health Baltimore USA; ^2^ “Soins et Sant” primaire CESP Centre for Research in Epidemiology and Population Health France; ^3^ Department of Community Health and Behavioural Sciences School of Public Health Makerere University Kampala Uganda; ^4^ Department of Epidemiology and Biostatistics School of Public Health Makerere University Kampala Uganda

## Abstract

Norms and beliefs toward contraception, both positive and negative, motivate contraceptive use; however, they have seldom been explored longitudinally in low‐ and middle‐income countries, limiting our understanding of their influence on contraceptive dynamics. We used PMA2020 Uganda national longitudinal data of reproductive aged women in 2018 (baseline) and 2019 (follow‐up) to explore discontinuation and switching among modern contraceptive users at baseline (n = 688) and contraceptive use at follow‐up among nonusers at baseline (n = 1,377). Multivariable simple and multinomial logistic regressions assessed the association of individual and community‐level contraceptive beliefs with contraceptive uptake, discontinuation and switching. One‐quarter of nonusers at baseline were using contraception at follow‐up, while 37 percent of users at baseline had discontinued and 28 percent had switched methods at follow‐up. The odds of contraceptive uptake were lower among women who strongly agreed that contraception impacted future fertility or caused conflict within a couple, relative to those who strongly disagreed (adjusted odds ratio (aOR): 0.7 and aOR: 0.6, respectively), but higher among women who strongly agreed that contraception preserved beauty (aOR: 1.6). Women who strongly agreed that it was acceptable to use contraception before having children were less likely to discontinue their method than those who strongly disagreed (adjusted relative risk ratio (aRRR): 0.5), though living in a community where more women agreed with this statement was associated with higher discontinuation (aRRR: 6.0). Family planning programs that promote positive beliefs toward family planning could improve contraceptive uptake and continuation. More research is needed to understand how contraceptive beliefs shape contraceptive decisions across the life course.

## BACKGROUND

Contraceptive use affords numerous health, social, and economic benefits. Many women who wish to avoid a pregnancy, however, are not using contraception. Satisfying unmet need for contraception could reduce maternal deaths by 29 percent and is one of the four pillars of the Safe Motherhood Initiative (Ahmed et al. 2012; Starrs 2006). In 2012, recognizing the potential of contraception to improve women's health and global development, the Family Planning 2020 (FP2020) initiative was launched with the goal of increasing access to modern contraceptives to 120 million additional women and girls in the world's 69 poorest countries by 2020. Despite the ambitious plans and commitments of governments, the world did not achieve the FP2020 goal (FP2020 2019). In the organization's midterm report, FP2020 highlighted several challenges that must be addressed to increase contraceptive coverage, including reducing high unmet need and addressing contraceptive discontinuation rates (FP2020 2019; “FP2020 Momentum at the Midpoint 2015–2016 | FP2020 Momentum at the Midpoint 2015–2016” 2016). Additionally, with increasing numbers of women adopting contraception, there is a shift toward understanding contraceptive dynamics, as a rising proportion of unmet need is attributed to “early” discontinuation rather than low rates of adoption. Factors contributing to contraceptive adoption and sustained use may be different, but such investigation requires longitudinal data and large population‐based cohorts.

There is increasing recognition that negative perceptions toward the safety and acceptability of contraception are widespread and represent a significant barrier to contraceptive use among women who want to avoid or delay pregnancy. Studies in a myriad of low‐income countries have identified that concerns about the general safety of contraceptive use (e.g., “contraceptives are not safe to use”) affect women's intentions to use and use of contraceptive methods (Machiyama et al. 2018; Huda et al. 2018; Machiyama and Cleland 2014), as do more specific considerations about safety, such as fear of infertility and bleeding changes. Fear of future infertility is a particularly pronounced and salient belief, especially for nulliparous women and women early in their reproductive years (Ghule et al. 2015; Sedlander et al. 2018; Ochako et al. 2015; Gueye et al. 2015; Schwarz et al. 2019). To date, few studies have explicitly distinguished women's fears of side effects from their own experience of side effects, making it difficult to disentangle the relationships each has on contraceptive dynamics. A small body of research does, however, demonstrate that the experience of certain side effects, specifically contraceptive‐induced menstrual changes, may contribute to fears of future infertility or cancer, particularly in sub‐Saharan Africa (SSA) (Schwarz et al. 2019; Polis, Hussain, and Berry 2018). The experience of side effects and the interpretation of them as expected and manageable or as potentially dangerous and life‐threatening is contextualized within a broader understanding of the safety and acceptability of contraception as a whole (Schwarz et al. 2019). Thus, understanding how specific contraceptive beliefs shape individual contraceptive behaviors is important to understand.

Beliefs about interpersonal and social consequences, such as spousal disapproval or social sanctions, are also associated with use. Evidence suggests that men and women in many SSA countries believe contraceptive use and subsequent delays in birth spacing can result in conflict within relationships and engender negative consequences within partnerships (Schwarz et al. 2019; Farmer et al. 2015; Bawah et al. 1999; Reiss et al. 2019; Kopp et al. 2018; Karp et al. 2020). Similarly, personal beliefs are informed by social interactions that convey broader societal expectations regarding fertility and contraceptive behaviors. Stephenson and colleagues demonstrate that women who live in communities where more women report approval of family planning (FP) are significantly more likely to use contraception in six countries in SSA, while Kincaid shows significant increases in contraceptive use as a result of a social network intervention in Bangladesh (Stephenson et al. 2007; Kincaid 2000). Both quantitative studies underscore the importance of community influences in shaping decision‐making about contraceptive use (Cialdini and Trost 1998). The notion that social norms, defined as informal rules and standards that are understood by members of a group, shape individual behavior is further supported by qualitative evidence from SSA where pronatalist social expectations—that is, the idea that women's childbearing and reproductive roles are paramount—seem to discourage contraceptive use (Sinai et al. 2019; Kane et al. 2016). Studies that have assessed community influences, operating as social norms, on contraceptive use have rarely explored contraceptive beliefs directly; instead, they have relied on statements that assess a general sense of approval (e.g., “Do you approve of family planning?”) aggregated at the community‐level or proxy measures of acceptability, such as the proportion of women in the community using family planning (Stephenson et al. 2007; Metheny and Stephenson 2017; Zimmerman et al. 2019). Measures of social norms and community beliefs that are defined by these broad definitions have made it particularly challenging for researchers to assess how much influence ideas shared at the community‐level have in women's individual contraceptive practices.

Although there is strong evidence of the important role that personal beliefs about the safety and acceptability of contraception play in individual contraceptive decision‐making, in addition to evidence that social norms at the community level also influence use, significant limitations to the literature remain. The majority of findings are based on cross‐sectional data or are generated from nonrepresentative samples. This limits our ability to determine if concerns affect uptake and continuation differently and limits the generalizability of findings. Few recent nationally representative longitudinal surveys include questions about specific beliefs toward contraception that can be assessed at the individual and community levels. Instead, many studies rely on nonspecific questions (Stephenson et al. 2007; Kaggwa, Diop, and Storey 2008; Cleland, Ndugwa, and Zulu 2011; DeRose et al. 2004) while most of the evidence exploring specific contraceptive beliefs has utilized qualitative methods and/or studied small samples, thereby limiting the generalizability of results to larger populations. Finally, the majority of research that has assessed specific beliefs has focused on negative beliefs around family planning, with a very limited body of evidence focused on understanding what women see as potential benefits to contraceptive use (Farmer et al. 2015; Duclos et al. 2019; Morse et al. 2014). As behavior is motivated by both positive and negative beliefs (Ajzen 1991), it is critical to understand not only the negative beliefs that may constrain behavior, but also the relevant positive beliefs that may motivate contraceptive use.

Our study, based on a nationally representative longitudinal study in Uganda, addresses many of the shortcomings outlined. Our objectives were to prospectively assess whether individual and community beliefs toward contraception were associated with changes in contraceptive practices over time, including: (1) adoption of a modern contraceptive method among sexually active nonusers, and (2) discontinuation of a modern contraceptive method among sexually active users.

## METHODS

### Study Site

Uganda has shown consistent growth in modern contraceptive use among women aged 15–49 years, from 21 percent in 2014 to 30 percent in 2018 (Makerere University, School of Public Health at the College of Health Sciences and Bill and Melinda Gates Institute for Population and Reproductive Health at the Johns Hopkins Bloomberg School of Public Health 2018). While injectables was the most common method used in 2018 (39 percent), implants have been growing in popularity and accounted for 20 percent of all use (Makerere University, School of Public Health at the College of Health Sciences and Bill and Melinda Gates Institute for Population and Reproductive Health at the Johns Hopkins Bloomberg School of Public Health 2018). Despite this increase in contraceptive use, unmet need for contraception and contraceptive discontinuation are prevalent in Uganda. As of 2018, unmet need for contraception was estimated at 21 percent of all women of reproductive age and 26 percent of married women (Makerere University, School of Public Health at the College of Health Sciences and Bill and Melinda Gates Institute for Population and Reproductive Health at the Johns Hopkins Bloomberg School of Public Health 2018). High rates of contraceptive discontinuation contribute to persistently high levels of unmet need. National estimates from the 2016 Demographic and Health Survey (DHS) indicate that 20 percent of intrauterine device (IUD) users, 17 percent of implant users, 36 percent of injectable users, and 46 percent of pill users discontinued in the first year of use, while still in need of protection against unintended pregnancy.

### Data

Performance Monitoring and Accountability 2020 (PMA2020) has conducted annual cross‐sectional surveys between 2014 and 2018 in Uganda. In the sixth cross‐sectional survey, fielded between April and May 2018, we modified the consent and design so that women who consented would be followed up to complete another interview one year later; the 2018 interview is hereafter referred to as the baseline survey. The follow‐up interviews were conducted from May to June 2019. PMA2020 is a multistage cluster, nationally representative survey of women aged 15–49. In the baseline survey, 110 Enumeration Areas (EAs) were selected using probability proportional to size sampling and all occupied households were enumerated. Forty‐four households were randomly selected within each EA, consented, and interviewed. All women aged 15–49 who were either usual members of the household or who slept in the household the night before were approached for interview, and interviewed face‐to‐face by a trained interviewer in one of eight local languages, if they consented to participate. Interviews were conducted within or nearby the respondents’ homes per respondent preference, and interviewers were trained to ensure auditory and visual privacy to the extent possible. On average, both the baseline and follow up interviews took 30 minutes. Baseline and follow‐up interviews covered similar topics including fertility intentions, contraceptive use and side effects, sexual activity and abortion. Further information on the design of PMA2020 surveys is available from www.pmadata.org and Zimmerman et al (2017).

Questionnaire items on beliefs about contraception were informed by PMA2020's Women's and Girl's Empowerment in Sexual and Reproductive Health qualitative study exploring women's decision‐making related to childbearing and contraception, including personal and community attitudes and beliefs about contraception (Karp et al. 2020; Wood et al. 2020; Moreau et al. 2020). Briefly, data from Uganda were reviewed to identify a range of prevalent beliefs that arose from semistructured in‐depth interviews and focus group discussions around both the benefits and drawbacks of using contraception. Questions were refined with interviewers during training and piloted among a sample of women aged 15–49 who lived in EAs that were not selected for PMA2020. There were no specific recommendations to wording based on the pilot tests.

A total sample of 4,288 women were interviewed in the baseline interview; the majority (95.5 percent; n = 4,095) of women agreed to participate in the follow‐up survey. At follow‐up, interviewers returned to the households of women who completed the baseline interview and re‐consented women to participate in the follow‐up interview. If women were no longer living in the original household but remained in the same EA, interviewers re‐contacted them by phone to set up an in‐person interview. We were able to relocate and successfully interview 2,755 of the original sample, resulting in a follow‐up rate of 67.0 percent.

We restricted the analytic sample to exclude women who at baseline reported one or more of the following: never being sexually active, being infertile, or using male or female sterilization (n = 477). We further excluded women who, at baseline, reported at baseline that they wanted another child either “soon” or within one year, as they would have limited motivation to either start or continue using contraception (n = 213). To assess the effect of beliefs on contraceptive uptake, we limited the sample to women who were not using a modern method, including women pregnant at baseline (n = 1,377 unweighted, n = 1,120 weighted). To assess contraceptive discontinuation and switching, we used only the observations from women who were using any modern method at baseline other than female sterilization (n = 688 unweighted, n = 870 weighted).

The study protocol was approved by Institutional Review Boards at the Makerere University School of Public Health in Kampala, Uganda, at the Bloomberg School of Public Health at Johns Hopkins University in Baltimore, USA, and at the Uganda National Council of Sciences and Technology.

### Dependent Variables

We examined two outcomes: adoption and continuation of a modern contraceptive method by comparing contraceptive use status at baseline and at follow‐up interview. Among women who were not using contraception at baseline, our outcome of interest was adoption of a modern contraceptive method (1—if women were nonusers at baseline and users at follow‐up; 0—if they remained nonusers in both surveys). Among women who were using a modern contraceptive method at baseline, our outcome of interest was continuation of a modern contraceptive method, defined in three categories, coded as continued use (0—using the same method at baseline and follow‐up), switched (1—using a different modern method at baseline and follow‐up), or discontinued (2—no longer using a modern contraceptive method or pregnant at follow‐up).

### Independent Variables

Six items measuring women's beliefs about contraception were asked at baseline. Three items assessed women's positive beliefs toward contraception, including: “if a woman uses family planning, she can have sex without worrying about pregnancy”; “a woman's beauty will last longer if she practices family planning”; and “it is acceptable for a woman to use family planning before she has children.” The other three items assessed women's negative beliefs about contraception, including: “it is unhealthy for women not to get periods when they are using injectables, pills, or implants”; “if a woman uses family planning, she may have a hard time getting pregnant when she wants to”; and “using family planning creates conflict in a couple.” Response options were structured using a Likert scale with options: “strongly agree,” somewhat agree,” “somewhat disagree,” and “strongly disagree.” Informed by findings of other studies, we decided to exclude a neutral category during data collection to reduce the chance that respondents would pick a neutral option when they did in fact have an opinion (Nowlis, Kahn, and Dhar 2002; Krosnick et al. 2002; Bishop 1987). In exploratory analyses, however, we found that few women chose the “somewhat agree” or “somewhat disagree” options and that the associations of interest were consistent between these two options. Thus, for analysis purposes, we recategorized “somewhat agree” and “somewhat disagree” into one “neutral” category.

Individual beliefs were further used to assess community‐level norms on modern contraceptive use, by averaging women's individual responses at the EA level. Specifically, for each belief item, we generated a variable that measured the percent of women in each EA who strongly agreed with each statement, removing each respondent's answer from the calculation to limit endogeneity. We measured the proportion of women who strongly agreed in order to remain consistent with our recategorization of “somewhat agreed” as a neutral category, and used responses from all women who participated in the baseline survey (n = 4,288) to generate the community‐level measures.

### Covariates

All analyses were adjusted for sociodemographic variables that have been identified as relevant to contraceptive use dynamics in previous literature, including age (categorical variable in five‐year age groups), marital status (binary variable indicating married/in‐union or not), residence (urban or rural), wealth (categorical variable of wealth quintiles), education (categorical variable indicating none, primary, or O‐level [equivalent to lower secondary] and above), parity (categorical variable of 0, 1–2, 3–4, or 5+ children), and fertility intentions. Fertility intentions were classified as either wanting another a child after one year, or wanting no more children. Variables that were specific to the analysis of uptake also included pregnancy status at baseline and previously ever using a modern method of contraception. When assessing continuation, discontinuation and switching, we included a binary variable indicating whether the respondent reported using a short‐acting (pill, injectable, male or female condom, lactational amenorrhea method (LAM), cycle beads, Emergency contraception (EC) or long‐acting (implant, IUD) method at baseline.

### Loss‐to‐Follow‐Up Weights

Because of potential bias from loss‐to‐follow‐up, we constructed an inverse propensity score by estimating a multivariate regression model with age, parity, marital status, schooling, wealth quintile, and residence as covariates. The predicted probability of loss‐to‐follow‐up was then multiplied by the original baseline individual female weight and its inverse was applied to re‐weight the follow‐up responses appropriately. Table A1 of the Appendix (in the Supporting Information) compares the weighted responses of the full sample of women in the baseline sample with those who completed follow‐up. After adjusting for the predicted probability of loss‐to‐follow‐up, there were no differences (p < 0.05) in the majority of baseline characteristics between all women who completed baseline interviews and those who were followed‐up one year later, including in the percentage of women using modern contraception. Only the percentage of women who wanted a child within a year was statistically significantly different between the two groups (15 percent among all women at baseline and 13 percent among women who were followed‐up).

### Analyses

Exploratory analyses assessed the frequencies of sample characteristics, women's agreement with each of the contraceptive beliefs questions, and contraceptive adoption, discontinuation and switching between baseline and follow‐up.

Among women who were not using modern contraception at baseline, we conducted bivariate logistic regressions to determine the unadjusted association between women's individual contraceptive beliefs and adoption of modern contraception at follow‐up. We then created a multivariable logistic regression to model adoption of contraception that included the woman's individual belief about contraception, the corresponding community‐level belief about contraception, and adjusted for sociodemographic variables.

Among women who were modern contraceptive users at baseline, we conducted a series of bivariate multinomial logistic regressions to determine the unadjusted associations between women's individual contraceptive beliefs with contraceptive discontinuation and switching, relative to continued use. We then created a multivariable, multinomial regression to model discontinuation and switching, including the woman's individual beliefs about contraception, and the corresponding community‐level belief, adjusting for sociodemographic variables.

All statistical tests in regression models adjusted for weighting, clustering effects due to multistage stratified cluster survey design of PMA2020, and differential lost‐to‐follow‐up, as previously described. Statistical significance was set at p < 0.05.

## RESULTS

### Descriptive Statistics

Table 1 shows the weighted baseline sample characteristics of the total sample and by baseline contraceptive use. The majority of women were married or living with a man as if married (72 percent), lived in rural areas (78 percent), had given birth at least once (86 percent), and had attained at least some primary schooling (91 percent). Though the sample was composed only of women who stated they wanted to delay childbearing for at least one year or have no more children, 61 percent of women were not using a modern method of contraception at baseline. Among nonusers of contraception in our sample, 23 percent were pregnant at baseline and over half had used previously used a method of modern contraception.

**TABLE 1:** Baseline characteristics of women, among all women and by contraceptive use status

	All	Nonusers	Users
	N = 2,065	N = 1,377	N = 688
	%	%	%
Age (mean)	28.9	29.0	28.7
Married or in‐union	72.4	70.1	76.8
Urban	21.9	20.7	24.3
Parity			
0	14.4	17.0	9.4
1–2	31.1	28.6	35.9
3–4	23.2	21.6	26.2
5+	31.3	32.8	28.5
Education			
None	9.4	11.4	5.5
Primary	56.8	59.5	51.6
O‐level+	33.9	29.1	42.9
Fertility intention			
Want in 1+ years	65.3	62.7	70.3
Want no more	34.7	37.3	29.7
Pregnant at baseline	14.9	22.7	–
Ever used a method of FP	68.9	52.6	–
Type of method			
None	60.5	100.0	–
Short‐acting	29.0	–	69.6
Long‐acting	10.4	–	30.4
Sex without worry			
Strongly disagree	5.3	6.5	3.2
Neutral	19.3	21.6	14.8
Strongly agree	75.4	71.9	82.0
Preserves beauty			
Strongly disagree	20.8	20.9	20.5
Neutral	29.7	31.1	27.0
Strongly agree	49.5	48.0	52.5
OK before children			
Strongly disagree	59.4	56.9	64.4
Neutral	16.6	18.0	13.8
Strongly agree	24.0	25.1	21.8
No period unhealthy			
Strongly disagree	12.5	11.8	14.0
Neutral	21.5	23.2	18.2
Strongly agree	66.0	65.0	67.8
Hard to get pregnant			
Strongly disagree	19.8	18.9	21.5
Neutral	33.1	34.2	31.0
Strongly agree	47.1	47.0	47.5
Causes conflict			
Strongly disagree	13.3	10.8	18.1
Neutral	30.9	31.7	29.4
Strongly agree	55.7	57.4	52.5
Contraceptive dynamics			
Continued nonuse	49.4	75.1	–
Adopted	16.4	24.9	–
Discontinued	12.7	–	37.0
Switched	9.7	–	28.3
Continued	11.9	–	34.6

### Beliefs toward Family Planning

At baseline, the majority of women (75 percent) strongly agreed that a woman who uses family planning can have sex without worrying about pregnancy and half (50 percent) strongly agreed that using family planning preserves a woman's beauty. More than half of women (59 percent) strongly disagreed that it was acceptable for a woman to use family planning before she had children. Almost half of women (47 percent) strongly agreed that a woman may have a hard time getting pregnant after using family planning and two‐thirds strongly agreed that it was unhealthy for women to not menstruate when using hormonal methods (66 percent). More than half of all women (56 percent) strongly agreed that use of family planning creates conflict within a couple.

Among all sexually active women who were not using contraception at baseline, 25 percent were using a modern method at follow‐up. Among those who were using contraception at baseline, 37 percent had discontinued, 35 percent were using the same method, and 28 percent had switched methods by follow‐up.

### Contraceptive Adoption

Table 2 shows the results of bivariate and multivariable models on the odds of contraceptive uptake at follow‐up. Women who strongly agreed that using contraception would make it harder to get pregnant later had significantly lower odds of adopting a modern method by follow‐up than women who strongly disagreed (aOR: 0.6 [0.4–1.0]). Women who were neutral or who strongly agreed that using family planning preserved beauty had higher odds of adoption relative to women who strongly disagreed with this statement (aOR: 1.7 [1.0–2.7] and aOR: 1.6 [1.0–2.6], respectively). Strongly agreeing that using contraception causes conflict in a couple was also significantly associated with lower odds of adoption (aOR: 0.6 [0.4–0.9]). Community‐level beliefs about contraception had no effect on individual women's likelihood of adopting a method.

**TABLE 2:** Unadjusted odds ratio of individual beliefs with contraceptive adoption and adjusted odds ratios of contraceptive adoption, after accounting for EA agreement and relevant sociodemographic characteristics

	Unadjusted	Adjusted
	OR (L–H 95% CIs)	OR (L–H 95% CIs)
Sex without worry		
Strongly disagree (ref)		
Neutral	0.52 (0.28–0.96)*	0.77 (0.4–1.5)
Strongly agree	0.50 (0.26–0.94)*	0.56 (0.32–1.01)^†^
EA agreement		1.19 (0.58–2.47)
Preserves beauty		
Strongly disagree (ref)		
Neutral	1.61 (1.04–2.49)*	1.66 (1.04–2.65)*
Strongly agree	1.47 (0.95–2.26)^†^	1.63 (1.03–2.59)*
EA agreement		0.92 (0.46–1.83)
OK before children		
Strongly disagree (ref)		
Neutral	1.09 (0.7–1.69)	1.06 (0.66–1.69)
Strongly agree	1.43 (0.87–2.38)	1.40 (0.86–2.29)
EA agreement		0.67 (0.22–2.03)
No period unhealthy		
Strongly disagree (ref)		
Neutral	1.20 (0.68–2.12)	1.05 (0.55–2.01)
Strongly agree	1.52 (0.87–2.66)	1.17 (0.66–2.1)
EA agreement		0.93 (0.44–1.96)
Hard to get pregnant		
Strongly disagree (ref)		
Neutral	0.93 (0.59–1.47)	0.96 (0.57–1.61)
Strongly agree	0.72 (0.48–1.08)	0.65 (0.41–1.00)*
EA agreement		1.5 (0.78–2.87)
Causes conflict		
Strongly disagree (ref)		
Neutral	0.71 (0.47–1.09)	0.78 (0.48–1.26)
Strongly agree	0.52 (0.37–0.74)***	0.60 (0.4–0.9)**
EA agreement		1.13 (0.51–2.51)

^*^p < 0.05, ^**^p < 0.01, ^***^p < 0.001, ^†^p < 0.10.

NOTE: Adjusted for age, marital status, wealth, education, parity, residence, fertility intention, pregnancy status at baseline, and previous use of a modern method of contraception.

The full adjusted models assessing contraceptive adoption between baseline and follow‐up, including the effects of background characteristics, are shown in Table A2 of the Appendix (in the Supporting Information). Across all models, age and marital status were associated with adoption. Women who were in the two wealthiest quintiles had significantly higher odds of contraceptive adoption than women in the poorest quintile.

### Contraceptive Discontinuation or Switching at Follow‐Up

Table 3 shows the results of the bivariate and multivariable multinomial models of the relative risk of discontinuation or switching contraception relative to continuing. In the adjusted models, women who strongly agreed that it was acceptable to use contraception before having children were significantly less likely to discontinue their method relative to women who strongly disagreed (aRRR: 0.5 [0.3–0.9]). Women who neither strongly agreed nor strongly disagreed with the statement that using contraception preserves beauty had a reduced risk of discontinuing relative to women who strongly disagreed (aRRR: 0.4 [0.2–0.9]). None of the individual beliefs about family planning were significantly associated with the risk of switching methods.

**TABLE 3:** Bivariate and multivariable relative risk ratios of contraceptive discontinuation and contraceptive switching relative to continued use by agreement with each FP belief among baseline users (reference group: strongly disagree)

	Discontinued		Switched	
	Unadjusted	Adjusted	Unadjusted	Adjusted
	RRR	RRR	RRR	RRR
Sex without worry				
Strongly disagree (ref)				
Neutral	0.73 (0.19–2.89)	0.78 (0.20–3.04)	1.26 (0.33–4.79)	1.32 (0.27–6.55)
Strongly agree	1.07 (0.35–3.27)	1.39 (0.45–4.29)	0.92 (0.29–2.91)	0.85 (0.19–3.83)
EA agreement		0.72 (0.17–3.04)		1.96 (0.36–10.72)
Preserves beauty				
Strongly disagree (ref)				
Neutral	0.37 (0.17–0.78)^**^	0.41 (0.19–0.85)^**^	0.54 (0.27–1.08)^†^	0.65 (0.32–1.31)
Strongly agree	0.49 (0.23–1.05)	0.61 (0.24–1.55)	0.67 (0.35–1.28)	0.63 (0.27–1.47)
EA agreement		0.55 (0.17–1.75)		1.15 (0.41–3.17)
OK before children				
Strongly disagree (ref)				
Neutral	1.01 (0.58–1.75)	1.03 (0.54–1.96)	1.14 (0.56–2.31)	1.17 (0.56–2.44)
Strongly agree	0.80 (0.46–1.41)	0.48 (0.25–0.92)^**^	1.1 (0.55–2.21)	0.74 (0.38–1.45)
EA agreement		6.03 (1.31–27.84)^**^		2.72 (0.47–15.6)
No period unhealthy				
Strongly disagree (ref)				
Neutral	0.91 (0.39–2.12)	0.78 (0.35–1.73)	0.61 (0.31–1.20)	0.56 (0.24–1.34)
Strongly agree	1.01 (0.56–1.80)	0.9 (0.47–1.72)	0.80 (0.44–1.46)	0.66 (0.34–1.28)
EA agreement		1.30 (0.34–4.96)		1.30 (0.36–4.66)
Hard to get pregnant				
Strongly disagree (ref)				
Neutral	0.81 (0.39–1.68)	0.81 (0.41–1.58)	0.63 (0.31–1.31)	0.74 (0.34–1.6)
Strongly agree	0.82 (0.42–1.61)	0.87 (0.46–1.65)	0.64 (0.30–1.36)	0.71 (0.27–1.84)
EA agreement		0.95 (0.38–2.37)		1.40 (0.39–4.99)
Causes conflict				
Strongly disagree (ref)				
Neutral	1.65 (0.77–3.54)	1.73 (0.79–3.77)	1.54 (0.75–3.17)	1.54 (0.68–3.51)
Strongly agree	1.6 (0.75–3.41)	1.59 (0.74–3.44)	1.30 (0.70–2.44)	0.96 (0.46–2.02)
EA agreement		0.94 (0.32–2.77)		1.57 (0.51–4.84)

^**^p < 0.01, ^†^p < 0.10.

NOTE: Adjusted for age, marital status, wealth, education, parity, residence, and fertility intention.

Living in a community with a greater percentage of women strongly agreeing that contraceptive use was acceptable before a first birth was significantly associated with greater discontinuation (aRR: 6.0 [1.3–27.8]). No other community norms about contraception were significantly associated with discontinuation or with switching.

The fully adjusted tables assessing contraceptive discontinuation and switching, including the effects of background characteristics, are shown in Tables A3 and A4 of the Appendix (in the Supporting Information), respectively. The relative risk of discontinuing was reduced by a factor of 0.3 for women using a long‐acting method at baseline relative to women using a short‐acting method (p < 0.01 across all models), but no other relationships were significant in the adjusted models. Relative to women with no education, women with primary education and women with secondary education were significantly more likely to have switched methods by follow‐up.

## DISCUSSION

Our objective was to assess whether a range of positive and negative beliefs about family planning, at both the individual and community levels, were associated with changes in women's contraceptive practices over a one‐year period in Uganda. Though the majority of women in Uganda had negative beliefs about family planning, we found that the effect of these beliefs on contraceptive dynamics varied significantly. Beliefs that contraceptive use causes infertility and concerns that using contraception creates conflict within a couple were widespread, even among contraceptive users. The ubiquitous nature of these beliefs, including among users, is consistent with other literature (Farmer et al. 2015; Machiyama et al. 2018; 2018; Sedlander et al. 2018; Ochako et al. 2015; Gueye et al. 2015; Polis, Hussain, and Berry 2018; Bawah et al. 1999); our findings demonstrate that such beliefs are a significant barrier to contraceptive adoption, though not continuation, in this population. We also find that positive beliefs about family planning favored adoption and decreased discontinuation, underscoring the need to highlight positive messaging around contraception in public health and communications campaigns, in addition to dispelling negative beliefs.

Three themes emerged as particularly salient to contraceptive practice: the benefit of maintaining beauty, preserving reproductive capacity, and avoiding conflict in relationships. Agreeing that contraception preserves a woman's beauty was positively related to adoption over the one‐year period and, in communities where more women strongly agreed with this statement, women were less likely to discontinue. This supports findings from qualitative research showing that women reported using contraception for birth spacing to avoid distorting their bodies, postpone aging, and maintain strong, feminine bodies (Duclos et al. 2019; Withers et al. 2015; Kibira et al. 2020). Additionally, qualitative studies among men have identified similar shared ideas, specifically that women who space their births by using contraception are healthier, stronger, and, therefore, more beautiful than women who do not use contraception and give birth more frequently (Withers et al. 2015; Kibira et al. 2020). Although the majority of positive messaging about the benefits of contraception have emphasized lowering the risk of maternal morbidity or mortality for both mother and child (Farmer et al. 2015; Duclos et al. 2019; Morse et al. 2014), these results highlight another potential positive message: that spacing children can allow women to maintain physical strength, health, and beauty. We found that this belief was influential at both the individual and community levels, indicating that promoting this message through social networks, in addition to individual messages in counseling, may be particularly effective at increasing contraceptive use among women who wish to prevent pregnancy. Kincaid's work in Bangladesh highlighted that social network approaches to addressing contraceptive ideation resulted both in higher adoption and lower discontinuation among women over a two‐year period (Kincaid 2000). Though more research is needed on which specific positive messages may be most effective in different settings, reinforcing the potential benefits of contraception to improved health and well‐being at multiple levels may be a means to improve contraceptive uptake and continuation among women at risk of unintended pregnancy.

The importance of preserving reproductive capacity was a central concern related to contraceptive behavior. There was widespread agreement that using family planning makes it difficult to get pregnant later. Almost half of the sample, both users and nonusers, strongly agreed with this statement, and the significant association seen between strongly agreeing with the statement and lower contraceptive adoption underscore the weight of these beliefs on women's reproductive behaviors. These findings align with research demonstrating that fear of future infertility is a major barrier to contraceptive use (Sedlander et al. 2018; Ochako et al. 2015), despite substantial evidence that reversible contraception does not affect women's ability to conceive once stopped (Barnhart and Schreiber 2009; Girum and Wasie 2018). Although the majority of studies have documented this qualitatively, we show here both the pervasiveness of these concerns in Uganda and the significant and specific barrier that they present to contraceptive adoption. In pronatalist cultures, such as Uganda, infertility is often associated with profound negative social and economic consequences (Sedlander et al. 2018; Schwarz et al. 2019; Dyer and Patel 2012; Patel 2016). The fear of future infertility thus likely reflects more than a fear of physical consequences, but also a complex fear of social and economic repercussions. Addressing fear of infertility in counseling, including through the provision of medically accurate and method‐specific information on the expected time to return to fertility after discontinuing contraception, is critical to women's comfort using contraception. Broader communications messaging targeting women, partners, families, and communities must also seek to address these harmful myths surrounding infertility, particularly given the high childbearing value placed on women in Uganda.

Concerns about preserving reproductive capacity are particularly important to address among adolescents and nulliparous women. Previous research has indicated that fear of infertility is a particularly pertinent concern among nulliparous and primiparous women (Sedlander et al. 2018; Ochako et al. 2015). Almost 60 percent of the sample strongly disagreed that it was acceptable to use contraception before a first birth and there is continued low use of contraception by nulliparous women, even when they wish to delay pregnancy. Our results indicate that these beliefs influence discontinuation, but the relationships are not straightforward. Strongly agreeing that it is acceptable to use family planning before having a child was associated with lower discontinuation, but living in a community where more women agreed with this statement was associated with higher discontinuation. Due to sample size limitations, we could not conduct additional analyses to explore how these relationships differed by parity, but it is likely that the influence of these beliefs differentially impacts women's contraceptive decisions before and after their first birth. As the goals of family planning programs increasingly shift toward meeting the changing needs of women from the time they become sexually active until menopause, additional research on how beliefs affect behavior differently over the reproductive life course is critically needed. Effective interventions and communication campaigns to improve contraceptive use may be very different for women entering their reproductive years relative to those who have finished childbearing; programs and policies would benefit from more nuanced research to inform these interventions and strategies.

Finally, concerns that contraceptive use caused conflict in a couple were common among both users and nonusers, again providing quantitative support to qualitative evidence that has consistently highlighted this concern and underscoring the extent to which this belief is shared (Farmer et al. 2015; Bawah et al. 1999). Agreeing that use of family planning causes conflict in a couple was significantly negatively related to adoption. Further research is necessary to understand whether beliefs are founded in previous experiences with partners and explore male beliefs toward contraception, including the potential positive roles of partners. Programs that aim to improve male engagement in reproductive health decision‐making should recognize that different messaging may be appropriate for couples at distinct stages of contraceptive use and over the reproductive life course. Finally, most research on partner influence in contraceptive use has been limited to its relation on nonuse and discontinuation, calling for a need for investigation into the role of partner support on adoption and switching patterns (Sarnak et al. 2021)

In terms of social influences, we found few associations between community norms about contraception (proxied by the percent of women who strongly agreed with each statement in the same EA) and individual women's use of contraception. This may not mean, however, that social norms do not influence contraceptive behavior. Rather, it is likely that the aggregated measure of the percentage of women who strongly agree with each statement is not an accurate measure of community norms as an aggregation of women's individual beliefs leave out other influential community members, such as men and older women, who contribute to the creation and perpetration of social norms. Unfortunately, however, we have no data from these populations. Though we found limited statistically significant relationships in our analysis, we believe that further exploration of the influence of shared beliefs on individual contraceptive use is warranted; such research would benefit by assessing beliefs among a wider range of individuals.

In addition to the limited measures of social norms, the sample size of users at baseline was relatively small and thus, limited our ability to detect statistically significant changes among this group (i.e., discontinuation and switching), despite large odds ratios that indicate beliefs about family planning are likely to influence sustained use. This particularly impacted our ability to analyze women's beliefs as originally intended (strongly disagree, somewhat disagreed, somewhat agree, and strongly agree). Though our models indicate that overall patterns were similar between those who somewhat disagreed and somewhat agreed with statements about contraception, it is likely that these groups are different. A larger sample size may also have allowed us to detect differences between nulliparous and parous women, an important area for future research as outlined above. Second, we were not able to account for events that occurred between the two time points and thus our measures of adoption, discontinuation, and switching do not account for change between the two rounds. We may, therefore, be misclassifying women, for example, nonusers at baseline who initially adopted contraception and then discontinued, or users at baseline who switched before discontinuing. The direction of bias that this introduces is unknown. We included a contraceptive calendar for this purpose, but we found that recall at the individual‐level, even over a one‐year period, was too inconsistent to correctly define continuation and adoption. Additionally, our sample had lower than expected retention at follow‐up. Though we were able to adjust for loss to follow‐up using inverse probability weights and create a sample that did not differ from the baseline group on observed variables once weighted, there may be unobserved differences between the original and follow‐up samples for which we cannot account. Finally, while we based the development of our measures on qualitative interviews that were conducted in the same population and that explored decision‐making about contraception, the qualitative research was not specifically designed for our research question. There may be additional beliefs about the safety and acceptability of family planning that would be relevant to explore further.

Despite these limitations, this study has many strengths. First, we used longitudinal data to demonstrate the differential predictive effect that individual beliefs and community‐level norms about contraception have on contraceptive dynamics. Previous literature has generally only been able to assess cross‐sectional associations, which limits our understanding of causality due to issues with temporality, and which obscures how beliefs affect the full range of contraceptive behaviors, as only use or nonuse can be assessed. Additionally, we measured both positive and negative beliefs toward contraception, demonstrating the importance of positive beliefs and contributing to an evidence base that is under‐researched. This focus on specific beliefs, rather than general approval or disapproval, also provided a more nuanced understanding of what beliefs are particularly influential on the decision to use, or discontinue contraception. Though understanding general levels of approval is important, these questions provide little guidance for policies and programs that aim to reduce unmet need for contraception and help women achieve their reproductive goals. Perceived benefits and drawbacks of contraceptive use are far‐ranging. Generating new evidence on the specific beliefs associated with different contraceptive behaviors, including the breadth and magnitude of these beliefs in a population, provides valuable data to inform programs.

## CONCLUSIONS

We found that a range of both positive and negative beliefs toward family planning was associated with women's contraceptive use dynamics in Uganda. Concerns about contraceptive use impacting future fertility and causing conflict in relationships were prevalent among users and nonusers of contraception and presented significant barriers to uptake, however, preserving beauty was identified as a strong motivation to use contraception. Identifying women's specific beliefs about contraception can help program and policy implementors develop and tailor messages that can motivate adoption and continuation, in addition to addressing negative beliefs, among women who want to avoid pregnancy. More research is needed, however, to determine how the influence of specific beliefs differ across the reproductive life course.

## CONFLICTS OF INTEREST

The authors have no conflicts of interest to disclose.

## ETHICS APPROVAL STATEMENT

The study protocol was approved by Institutional Review Boards at the Makerere University School of Public Health in Kampala, Uganda, at the Bloomberg School of Public Health at Johns Hopkins University in Baltimore, USA, and at the Uganda National Council of Sciences and Technology.

## Supporting information

Supporting Information
Appendix

## Data Availability

All data used in this publication are publicly available at https://www.pmadata.org/data/available‐datasets/request‐access‐datasets. Citations for the data are Makerere University, School of Public Health at the College of Health Sciences and The Bill & Melinda Gates Institute for Population and Reproductive Health at The Johns Hopkins Bloomberg School of Public Health. Performance Monitoring and Accountability 2020 (PMA2020) Survey Round 6, PMA2018/Uganda‐R6. 2018. Uganda and Baltimore, Maryland, USA and Makerere University, School of Public Health at the College of Health Sciences and The Bill & Melinda Gates Institute for Population and Reproductive Health at The Johns Hopkins Bloomberg School of Public Health. Performance Monitoring and Accountability 2020 (PMA2020) Round 6 Follow‐up Household and Female Survey, PMA2019/Uganda‐R6FU. 2019. Uganda and Baltimore, Maryland, USA. doi: 10.34976/9×2b‐nd72
