# Supplementary material for: Family Planning Beliefs and Their Association with Contraceptive Use Dynamics: Results from a Longitudinal Study in Uganda
Source: Stud Fam Plann. 2021 May 20;52(3):241–58. doi: 10.1111/sifp.12153 (PMC9290856; doi:10.1111/sifp.12153)
Supplement: Supplementary file 1 — Supporting Information Appendix [file SIFP-52-241-s001.docx]

APPENDIX T1 Comparison of weighted baseline and panel sample characteristic

APPENDIX T2 Unadjusted and adjusted odds ratios of contraceptive use at follow-up

*p<.05. **p<.01. ***p<.001. †p<.10

APPENDIX T3 Bivariate and multivariable relative risk ratios of contraceptive discontinuation relative to continued use

*p<.05. **p<.01. ***p<.001. †p<.10

APPENDIX T4 Bivariate and multivariable relative risk ratios of contraceptive switching relative to continued use

*p<.05. **p<.01. ***p<.001. †p<.10
